# MAINTAINING PERSONHOOD AND AUTONOMY: A GROUNDED THEORY OF COMMUNITY-BASED HOSPICE CARE TRANSITIONS

**DOI:** 10.1093/geroni/igad104.2367

**Published:** 2023-12-21

**Authors:** Catherine Mann, Suzanne Sullivan, Yu-Ping Chang

**Affiliations:** State University of New York at Buffalo, School of Nursing, Buffalo, New York, United States; SUNY Upstate Medical University, Syracuse, New York, United States; University at Buffalo, The State University of New York, Buffalo, New York, United States

## Abstract

Supporting care transitions as decline occurs for patients facing serious illness and their families necessitates ongoing identification of person-centered, family-oriented goals. However, little is known about the process patients and caregivers undergo when making decisions while transitioning to home hospice care. Using a Straussian grounded theory approach, we explored processes patients and caregivers use when transitioning from cure- to comfort-focused care. Data were collected through eight interviews of patients, patient and caregiver dyads, and hospice nurses (n=11). Participants were recruited from hospice and palliative care agencies serving three counties in Western New York. Interviews were recorded and transcribed verbatim. Data were analyzed using constant comparative method. Our results generated a theory grounded in the process of maintaining personhood and autonomy (e.g., changing roles, losing sense of self, wanting to be heard, wanting privacy) while making decisions to transition to hospice care. This process includes five contemporaneous steps: Shopping for the right services (e.g., recognizing existing care limitations, overcoming systemic barriers), Involving caregivers (e.g., acting as an advocate, filling other family members in, accepting the patient’s decisions), Navigating the business of dying while living (e.g., dealing with legalities, planning for the end), Processing and expressing emotions (e.g., fear, anger, trauma, loss), and Seeking input as health declines/recognizing futility (e.g., pursuing a diagnosis, validating what is happening with the nurse and physician, recognizing options). The process identified in the study reveals important mechanistic targets for the development of interventions that promote patient-centered hospice care transitions in the home setting.

